# Supplementary material for: Spatiotemporal computations in the insect celestial compass
Source: Nat Commun. 2025 Mar 22;16:2832. doi: 10.1038/s41467-025-57937-w (PMC11929787; doi:10.1038/s41467-025-57937-w)
Supplement: Supplementary file 1 — Supplementary Information [file 41467_2025_57937_MOESM1_ESM.pdf]

Supplementary Information

Spatiotemporal computations in the insect celestial compass

Evripidis Gkanias[1*] and Barbara Webb[1]

[1]School of Informatics, University of Edinburgh, Edinburgh, UK
[*]Corresponding author: ev.gkanias@gmail.com

Supplementary Text S1. Alignment of hour-angle to solar noon using the day length.

To align the hour angle (ω) at solar noon, we must estimate the duration between sunrise and sunset (day length, T_L). Then we can shift a sinusoid aligned with the sunrise (based on the zeitgeber time, t_z) by half the day's length to align it with the solar noon. This can be done either by subtracting the angles inside the sinusoid, or by using a trigonometric property to rotate the cosine and sin components of the hour-angle by the day length as

$$\omega_N(t) = -\cos\left((t_z - \frac{T_L(t)}{2})\,15^\circ\right) = \sin(7.5^\circ\,T_L(t))\,\sin(t_z\,15^\circ) - \cos(7.5^\circ\,T_L(t))\,\cos(t_z\,15^\circ),$$
$$\omega_E(t) = -\sin\left((t_z - \frac{T_L(t)}{2})\,15^\circ\right) = \sin(7.5^\circ\,T_L(t))\,\cos(t_z\,15^\circ) - \cos(7.5^\circ\,T_L(t))\,\sin(t_z\,15^\circ).$$

We consider it questionable whether neurons can internally subtract angles, but biologically plausible that they implement a trigonometric identity, as discussed elsewhere in the paper.

Supplementary Text S2. Smoothing temporal responses.

We managed to smooth the day length estimates by using a second differential equation with a long time-constant ($\tau'_L = 360\,\mathrm{h}$),

$$\tau'_L \frac{\mathrm{d}T'_L}{\mathrm{d}t} = T_L(t) - T'_L.$$

The above second-order processing smooths the estimates so that they now follow the pattern of changing day length more accurately throughout the year (Supplementary Fig. S2a and c-f, black lines; also smaller variance compared to grey boxes in Supplementary Fig. S2b). This suggests that a sequence of dynamic processes can smooth the responses of periodically activated neurons. Despite the lower variance of the errors, the median error increases due to the time delay introduced by low pass filtering. We can fix this problem and centre the estimated pattern at its actual day-length curve (orange dashed line) using the same principles as in Supplementary Text S1.

Supplementary Text S3. Day length given the solar declination and geometric latitude.

Although the day length (T_L) might look similar to the solar declination (δ), they differ. Solar declination is a property that describes the relationship between the earth's rotation axis and the sun, and it is independent of the geometric latitude of the observer on Earth. The day length is a function of the solar declination and the geometric latitude (ϕ) and therefore depends on both. Their relationship can be described as

$$T_L = \frac{2}{15^\circ}\,\cos^{-1}(-\tan(\delta)\tan(\phi)).$$

Using the above equation we can estimate the day length, solar declination or latitude using the other two terms as

$$\delta = \tan^{-1}(-\frac{\cos(7.5^\circ\,T_L)}{\tan(\phi)}),$$
$$\phi = \tan^{-1}(-\frac{\cos(7.5^\circ\,T_L)}{\tan(\delta)}).$$

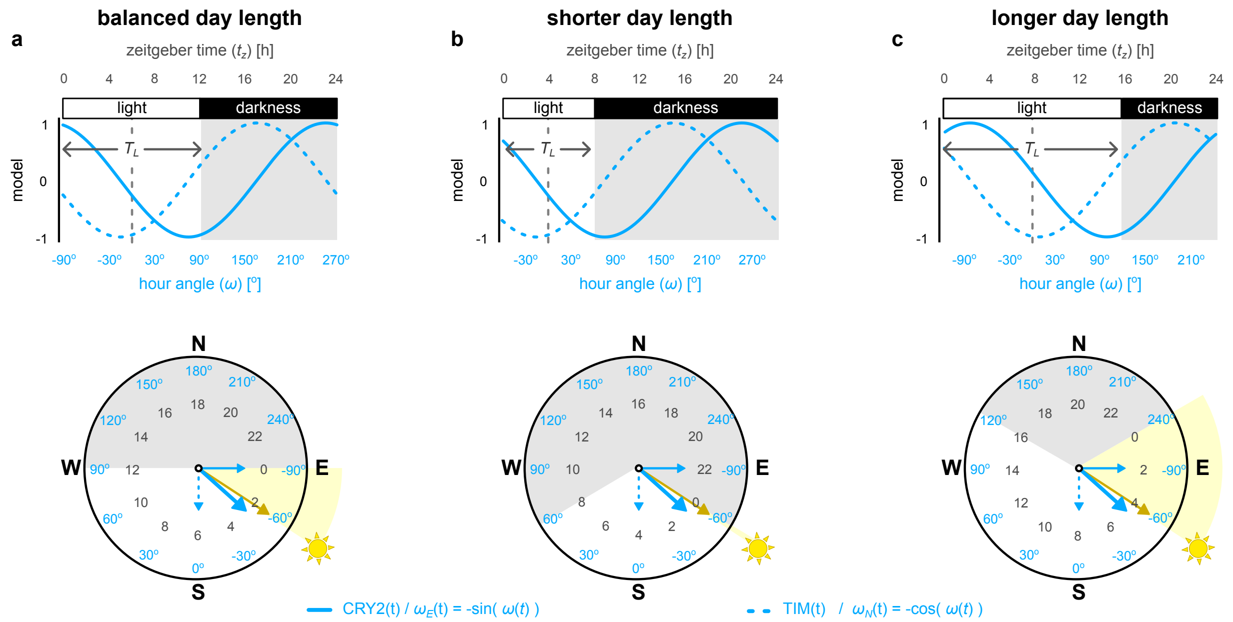

Supplementary Figure S1. The effect of the day length on hour angle. Two sinusoids represent the hour angle (ω) centred at solar noon. Although the pace of the sinusoids is given by the zeitgeber time (t_z), their phase is determined by the day length (T_L), which is illustrated for (**a**) balanced day and night, (**b**) shorter day than night, and (**c**) longer day than night. Top: the modelled mRNA levels of type 2 cryptochrome (Cry2) and timeless (Tim) proteins as cosine and sine functions of zeitgeber time. Note the shift in the phase of the sinusoids to align with the solar noon (dash vertical line). Bottom: The hour angle, encoded by combining Cry2 and Tim proteins, is an arrow that rotates clockwise (CW) and completes a full circle daily. The arrow should point east six hours before the solar noon (rather than at sunrise as assumed in previous models) to approximate the expected solar azimuth. N: north, E: east, S: south, W: west.

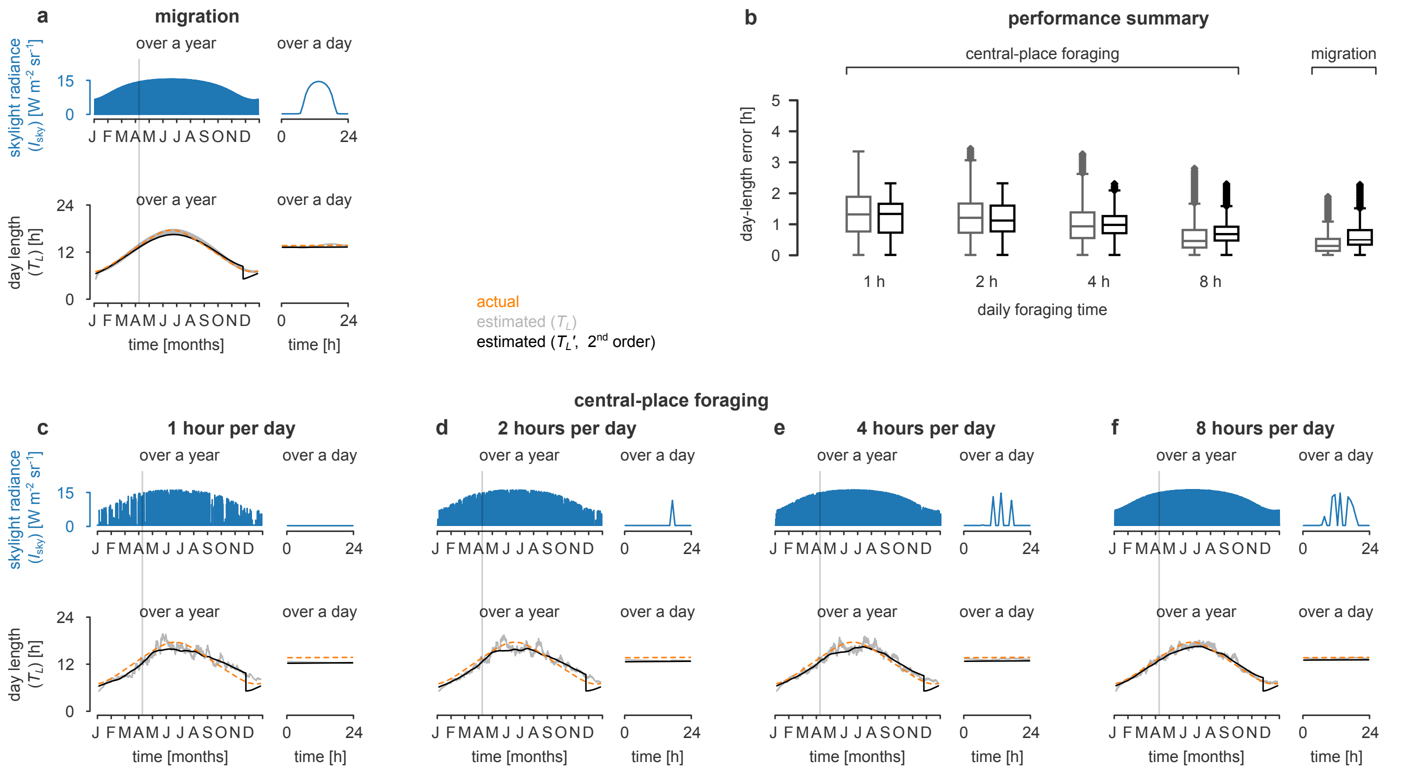

Supplementary Figure S2. Simulated estimation of day length. We fitted the dynamic equation for the day length (T_L) using the overall blue skylight irradiance as input. We simulated the skylight irradiance using the 'skylight' Python package. We then placed an insect at Edinburgh (55.9533N, 3.1883W) and performed (**a**) a migration experiment where the insect has unlimited exposure to daylight, or (**c**)-(**f**) different foraging patterns of 1-8 hours per day, where the insect is in the darkness for the rest of the time. The top panels show the blue light irradiance for the calendar year 2024 (left) and the date April 7, 2024 (right; highlighted with grey on the left plot). The bottom plots show the actual (orange dashed line) and estimated day length by integrating the skylight irradiance (grey and black solid lines). We report two estimates of the day length: one using a single dynamical equation (T_L; grey line), and one using a second dynamical equation in series (T_L'; black line). The letters on the horizontal axis represent the months' names. (**b**) Performance summary of the grey and black lines for the different conditions. Grey and black boxes summarise the error in the day length estimations of the grey and black lines, respectively. Each box shows the quartiles of the data ($n = 8,784$ samples span over the year 2024; 366×24). The whiskers extend to show the rest of the distribution except for points that exceed 1.5× the inter-quartile range, which are marked as outliers.

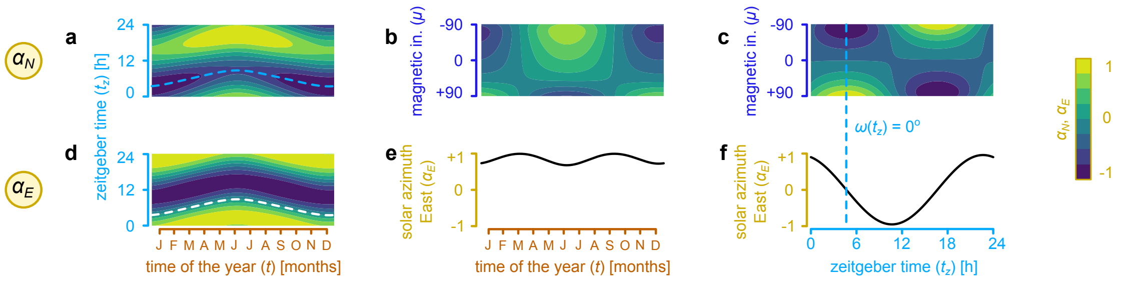

Supplementary Figure S3. Values of the solar azimuth components based on the different variables. The heatmaps represent the values of the north-most component of the solar azimuth based on (**a**) the time of the year (horizontal) and zeitgeber time (vertical), (**b**) the time of the year (horizontal) and the magnetic inclination (vertical), and (**c**) the zeitgeber time (horizontal) and the magnetic inclination (vertical). (**d**), (**e**), and (**f**) show the values of the east-most component of the solar azimuth based on the same variables. As the east-most component does not depend on the magnetic inclination, (**e**) and (**f**) omit this axis and show a line plot of the east-most component as a function of the time of the year and the zeitgeber time respectively.

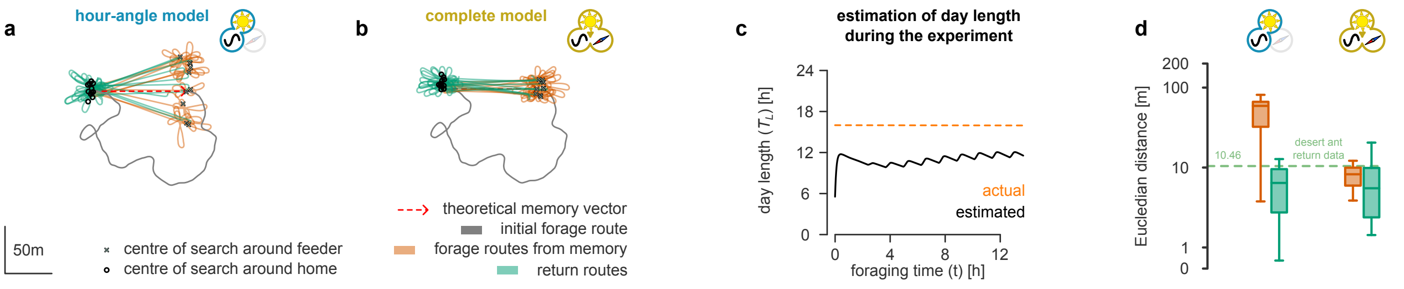

Supplementary Figure S4. Simulated central-place foraging routes using the estimated day length. The simulated insects search for food and store the food location after finding it. Then they return to their nest. They try to repeat foraging every hour and return to their nest before sunset, using (**a**) the hour-angle and (**b**) complete models. (**c**) Estimated (black solid line) and actual day length (orange dashed line) during the experiment. (**d**) Euclidean distance (m) of the search centroid from the feeder (red) or the nest (green) using the two models. The green dashed line indicates the desert ants' foraging error based on the equation $y = 2 \cdot 10^{-5}x^2 + 0.083x + 1.96\,\text{m}$, where x is the foraging distance [1]. Each box shows the quartiles of the data ($n = 9$ samples span over Aug 2, 2024; the initial foraging route is excluded). The whiskers extend to show the rest of the distribution except for points that exceed 1.5× the inter-quartile range, which are marked as outliers. There is 20 % added compass and processing noise in all results.

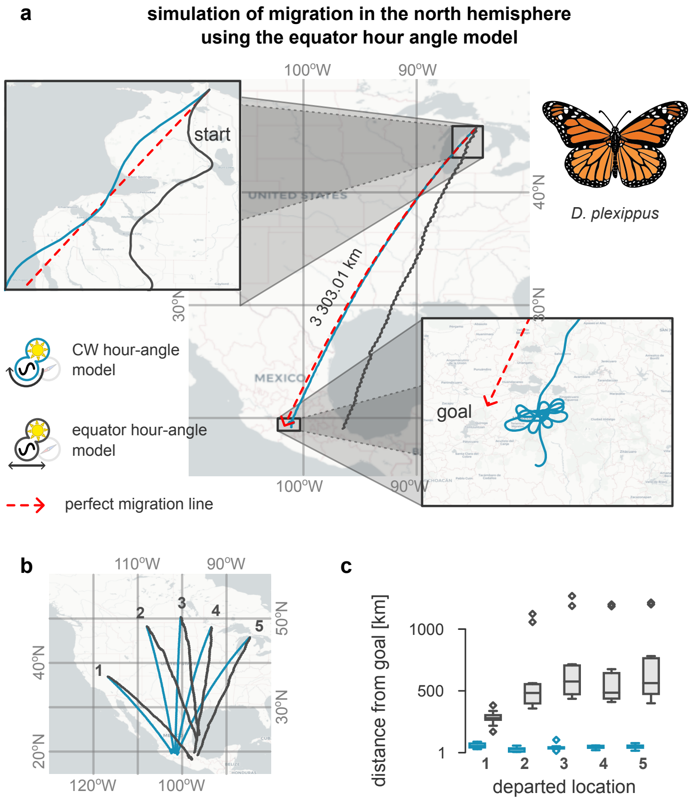

Supplementary Figure S5. Simulations of butterfly migrations using the equator hour-angle model (**a**) Simulation of a monarch butterfly (*Danaus plexippus*) during its autumn migration, using the clockwise (CW; blue) and equator hour-angle models (grey). The red dashed arrow illustrates the straight line between the start and goal locations. (**b**) Similar simulations, starting from five different locations across the Canadian border and California. (**c**) The Haversine distance (km) of the butterfly search centroid from the target location for each model and starting location. Each migration starts with small differences in location (±5°) or departure time (±72 h). There is 20 % added compass and processing noise in all results. Each box shows the quartiles of the data ($n = 10$ samples). The whiskers extend to show the rest of the distribution except for points that exceed 1.5× the inter-quartile range, which are marked as outliers. All maps were drawn with permission using the Contextly Python package with the CartoDB provider option.

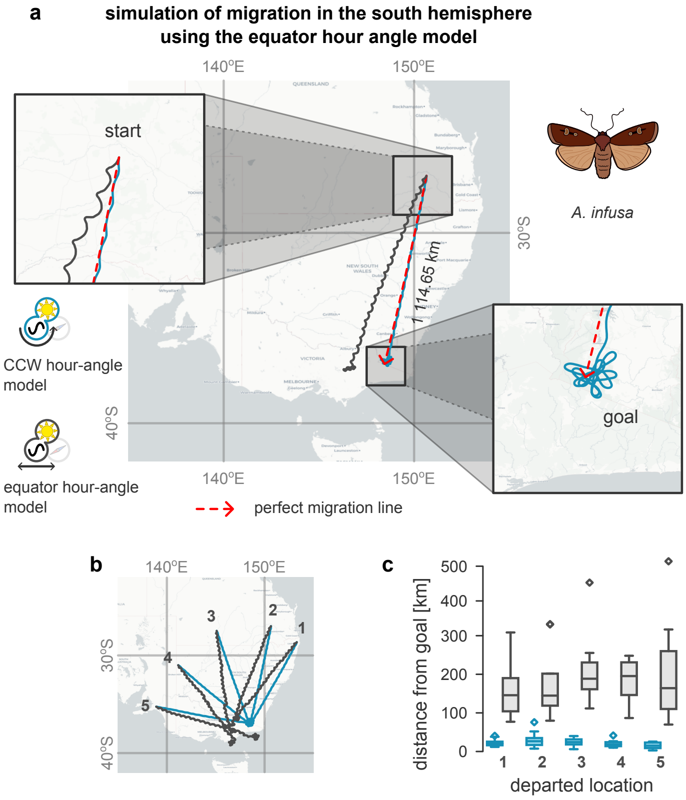

Supplementary Figure S6. Simulations of moth migrations using the equator hour-angle model
(**a** Simulation of a Bogong moth (*Agrotis infusa*) during its autumn migration, using counterclockwise (CCW; blue) and equator hour-angle models (grey). The red dashed arrow illustrates the straight line between the start and destination locations. (**b**) Similar simulations, starting from five different locations in Australia. (**c**) The Haversine distance (km) of the moth search centroid from the target location for each model and the starting location. Each migration starts with small differences in location ($\pm 5°$) or departure time ($\pm 72\,\text{h}$). There is $20\,\%$ added compass and processing noise in all results. Each box shows the quartiles of the data ($n = 10$ samples). The whiskers extend to show the rest of the distribution except for points that exceed $1.5\times$ the inter-quartile range, which are marked as outliers. All maps were drawn with permission using the Contextly Python package with the CartoDB provider option.

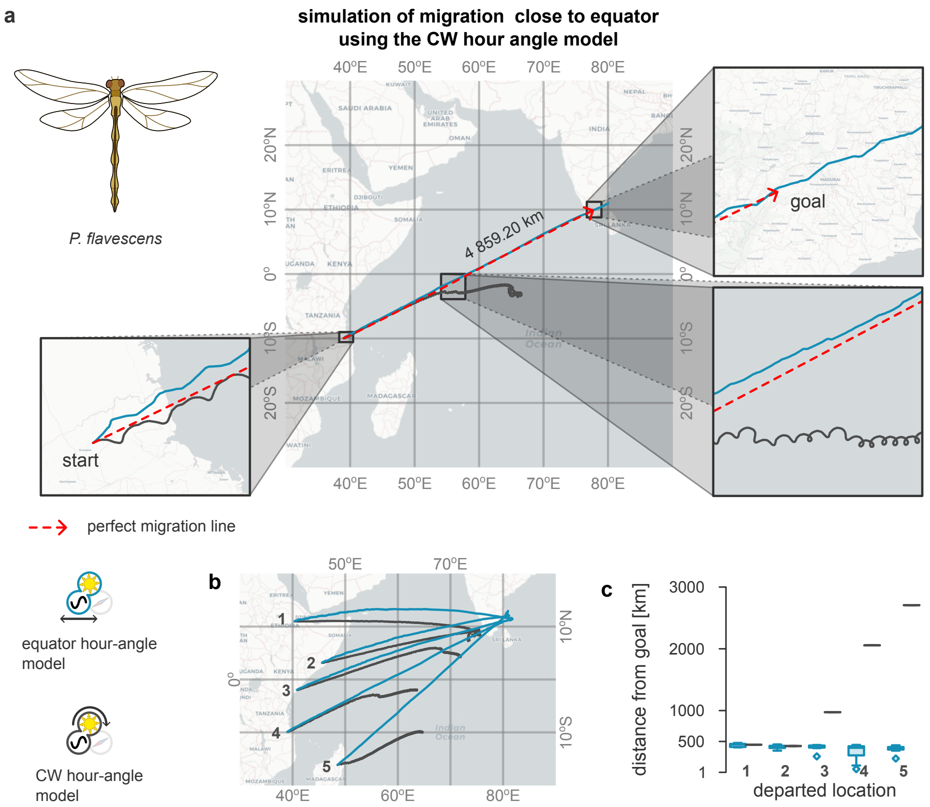

Supplementary Figure S7. Simulations of dragonfly migrations using the CW hour-angle model
(**a**) Simulation of a globe skimmer dragonfly (*Pantal flavescens*) during its spring migration, using the equator (blue) and clockwise (CW) hour-angle models (grey). The red dashed arrow illustrates the straight line between the start and destination locations. (**b**) Similar simulations, starting from five different locations in Australia. (**c**) The Haversine distance (km) of the dragonfly search centroid from the target location for each model and starting location. Each migration starts with small differences in location ($\pm 5°$) or departure time (± 72 h). There is 20 % added compass and processing noise in all results. Each box shows the quartiles of the data ($n = 10$ samples). The whiskers extend to show the rest of the distribution except for points that exceed $1.5\times$ the inter-quartile range, which are marked as outliers. All maps were drawn with permission using the Contextly Python package with the CartoDB provider option.

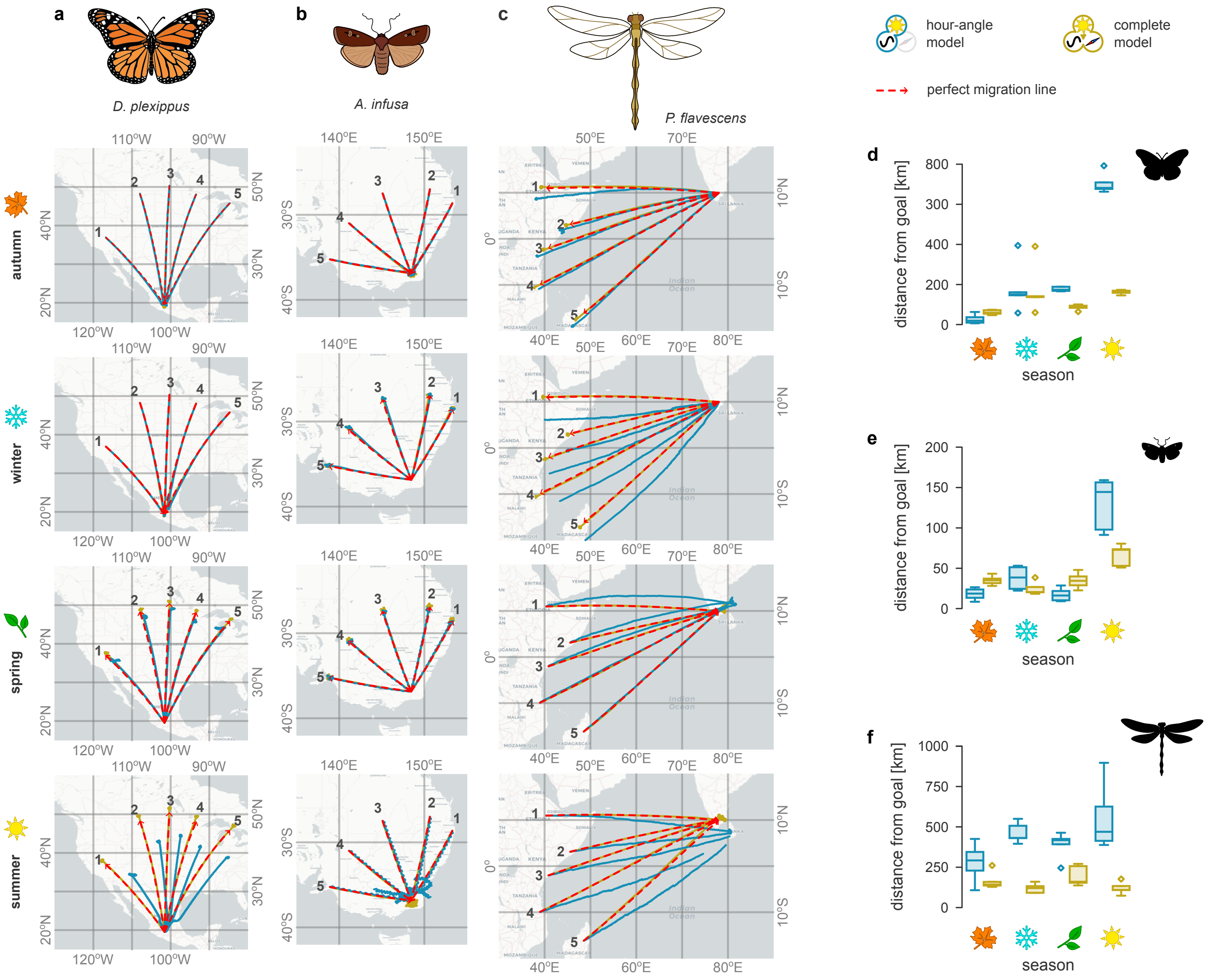

Supplementary Figure S8. Simulations of migrations in different seasons
(**a**) Simulation of monarch butterflies (*Danaus plexippus*) during migrations in different seasons, using the clockwise hour-angle (blue) and complete models (yellow). The red dashed arrow illustrates the straight line between the start and destination points. Migrations in autumn and winter start from the Canadian border or California and end in Mexico. Migrations are reversed for spring and summer. (**b**) Similar simulation for Bogong moths (*Agrotis infusa*). Migrations in summer and autumn start from several places in Australia and end on Mount Bogong. Migrations are reversed for winter and spring. (**c**) Similar simulation of a globe skimmer dragonfly (*Pantal flavescens*). Migrations in spring and summer start from the eastern African coast and end in India. Migrations are reversed for autumn and winter. (**d**) Haversine distance (km) of the search centroid from the desired destination of butterflies, conditioned to season and model. Each box shows the quartiles of the data ($n = 10$ samples). Variation comes from perturbations in the starting location ($\pm 5°$) and time (± 72 h). There is also 20 % added compass and processing noise in all results. The whiskers extend to show the rest of the distribution except for points that exceed 1.5× the inter-quartile range, which are marked as outliers. Similar Haversine distance for (**e**) the moths and (**f**) dragonflies. All maps were drawn with permission using the Contexty Python package with the CartoDB provider option.

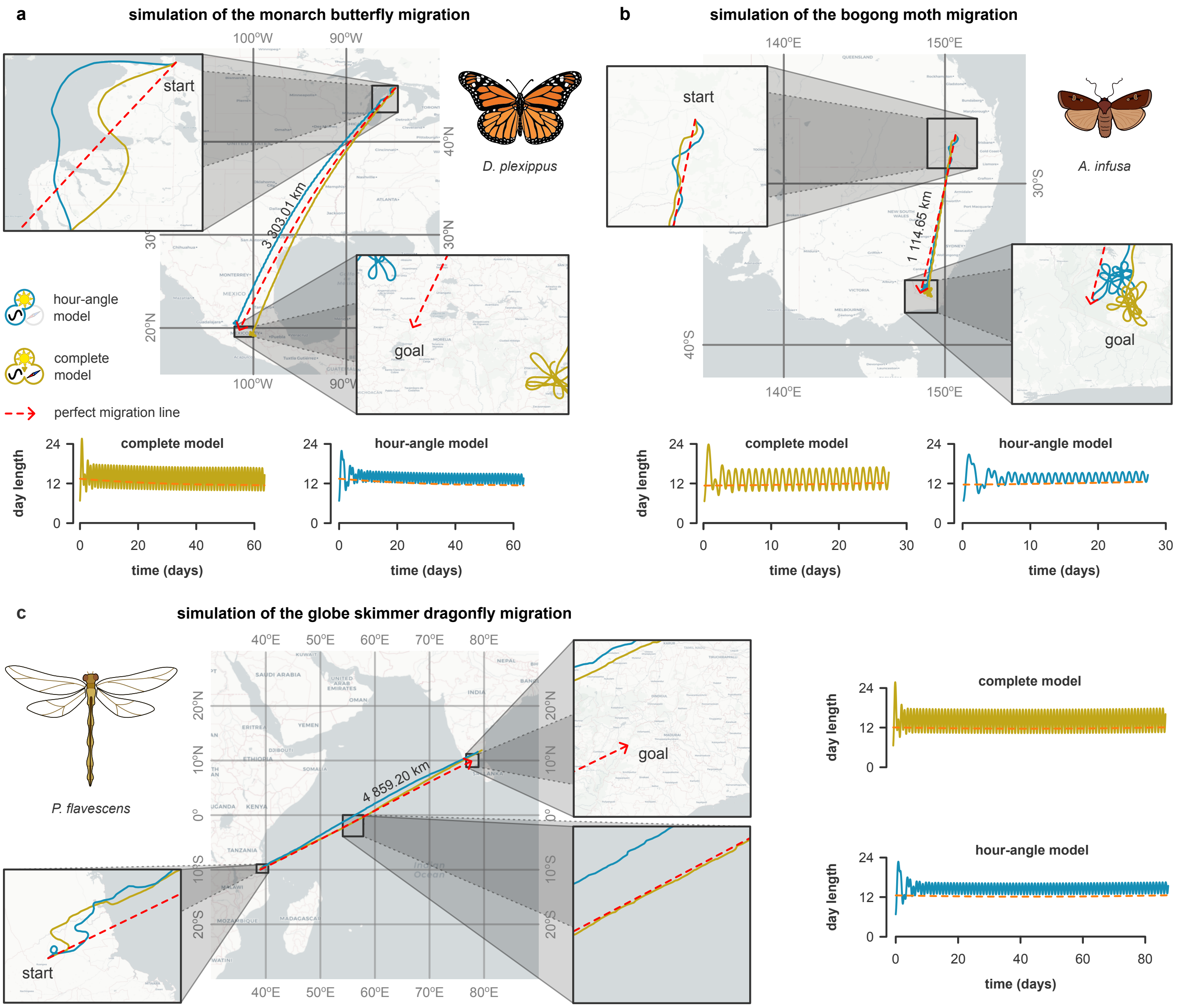

Supplementary Figure S9. Simulations of insect migrations using the estimated day length
(**a**) Simulation of a monarch butterfly (*Danaus plexippus*) during its autumn migration, using the hour-angle (blue) and complete models (yellow). The red dashed arrow illustrates the straight line between the start and destination locations. The estimated (yellow: complete; blue: hour angle) and actual day length (orange dashed line) during the migration are shown at the bottom of the panel. (**b**) Similar simulation for the Bogong moth (*Agrotis infusa*) during its autumn migration. (**c**) Simulation of a globe skimmer dragonfly (*Pantal flavescens*) during its spring migration. There is 20 % added compass and processing noise in all results. All maps were drawn with permission using the Contextly Python package with the CartoDB provider option.

Supplementary Table S1. Optimised parameters for the day length.

condition	foraging time (h)	τ_L (d)	a ($W^{-1}m^2sr$)	β (d)
central-place	1	12.08	14.56	6.55
foraging	2	10.22	7.94	6.02
	4	8.44	3.97	5.81
	8	6.33	2.14	5.35
migration	—	4.36	1.46	5.17

τ_L: time-constant for day length; a: gain of the skylight irradiance; β: time-constant bias.

Supplementary Table S2. Top input cell types to DN1p$_B$ neurons.

name	synapses	partners	NT type	hemisphere	input
SLP064	108 (28 %)	1 (4 %)	Glu (65 %)	right	MTe06 (76 / 29 %)
SLP364	67 (18 %)	2 (8 %)	Glu (73 %)	right	MTe06 (69 / 31 %)
SMP523	50 (13 %)	6 (24 %)	ACh (%)	left	CB3508 (12 / 36 %)
SLP270	43 (11 %)	2 (8 %)	ACh (87 %)	right	VP4+_vPN (193 / 20 %)
SMP529	20 (5 %)	1 (4 %)	ACh (60 %)	right	AVLP594 (86 / 14 %)
SLP207	13 (3 %)	1 (4 %)	GABA (84 %)	right	CL317 (79 / 17 %)
CB3536	12 (3 %)	1 (4 %)	GABA (50 %)	right	FS4A (55 / 36 %)
MeTu4	10 (3 %)	2 (8 %)	ACh (91 %)	right	Tm8a (27 / 51 %)
SLP001	10 (3 %)	1 (4 %)	Glu (73 %)	right	LHPV3c1 (29 / 18 %)
CB0113	9 (2 %)	1 (4 %)	ACh (39 %)	right	—

NT: neurotransmitter, SLP: superior lateral protocerebrum, SMP: superior medial protocerebrum, Me: medula, Tu: tubercle, LH: lateral horn, Glu: glutamate, ACh: acyl chloride, GABA: gamma-aminobutyric acid. Data from FlyWire[29].

Supplementary Table S3. Top output cell types from DN1p$_B$ neurons.

name	synapses	partners	NT type	hemisphere	output
TuTuBb	130 (19 %)	2 (5 %)	GABA (57 %)	right/left	MeTu2 (418 / 51 %)
TuBu01b	126 (19 %)	3 (8 %)	ACh (95 %)	right	ER4m/ER5 (244 / 71 %)
TuBu01a	82 (12 %)	2 (5 %)	ACh (95 %)	right	ER4m (301 / 78 %)
SLP463	52 (8 %)	3 (8 %)	Ser (78 %)	right	SMP505 (9 / 60 %)
CB071	45 (7 %)	2 (5 %)	Glu (71 %)	right	SLP286b (28 / 27 %)
SMP373	36 (5 %)	2 (5 %)	ACh (80 %)	right	SMP527 (78 / 28 %)
TuBu07	31 (5 %)	3 (8 %)	ACh (89 %)	right	ER3w (215 / 67 %)
CB1407	18 (3 %)	2 (5 %)	Glu (71 %)	right	SMP505 (41 / 50 %)
CB2517	16 (2 %)	2 (5 %)	Glu (77 %)	right	SMP505 (39 / 45 %)
CB1416	15 (2 %)	1 (2 %)	Glu (65 %)	right	LHPV5h2 (44 / 26 %)

NT: neurotransmitter, SLP: superior lateral protocerebrum, SMP: superior medial protocerebrum, Me: medula, Tu: tubercle, Bu: bulb, ER: ring neurons, LH: lateral horn, Glu: glutamate, ACh: acyl chloride, GABA: gamma-aminobutyric acid, Ser: serotonin. Data from FlyWire[29].

References

[1] Huber, R. & Knaden, M. Egocentric and geocentric navigation during extremely long foraging paths of desert ants. *Journal of Comparative Physiology A* **201**, 609–616 (2015).
